# Supplementary material for: Connexin 41.8 governs timely haematopoietic stem and progenitor cell specification
Source: Biol Open. 2025 Aug 12;14(8):bio062118. doi: 10.1242/bio.062118 (PMC12381925; doi:10.1242/bio.062118)
Supplement: Supplementary information [file biolopen-14-062118-s1.pdf]

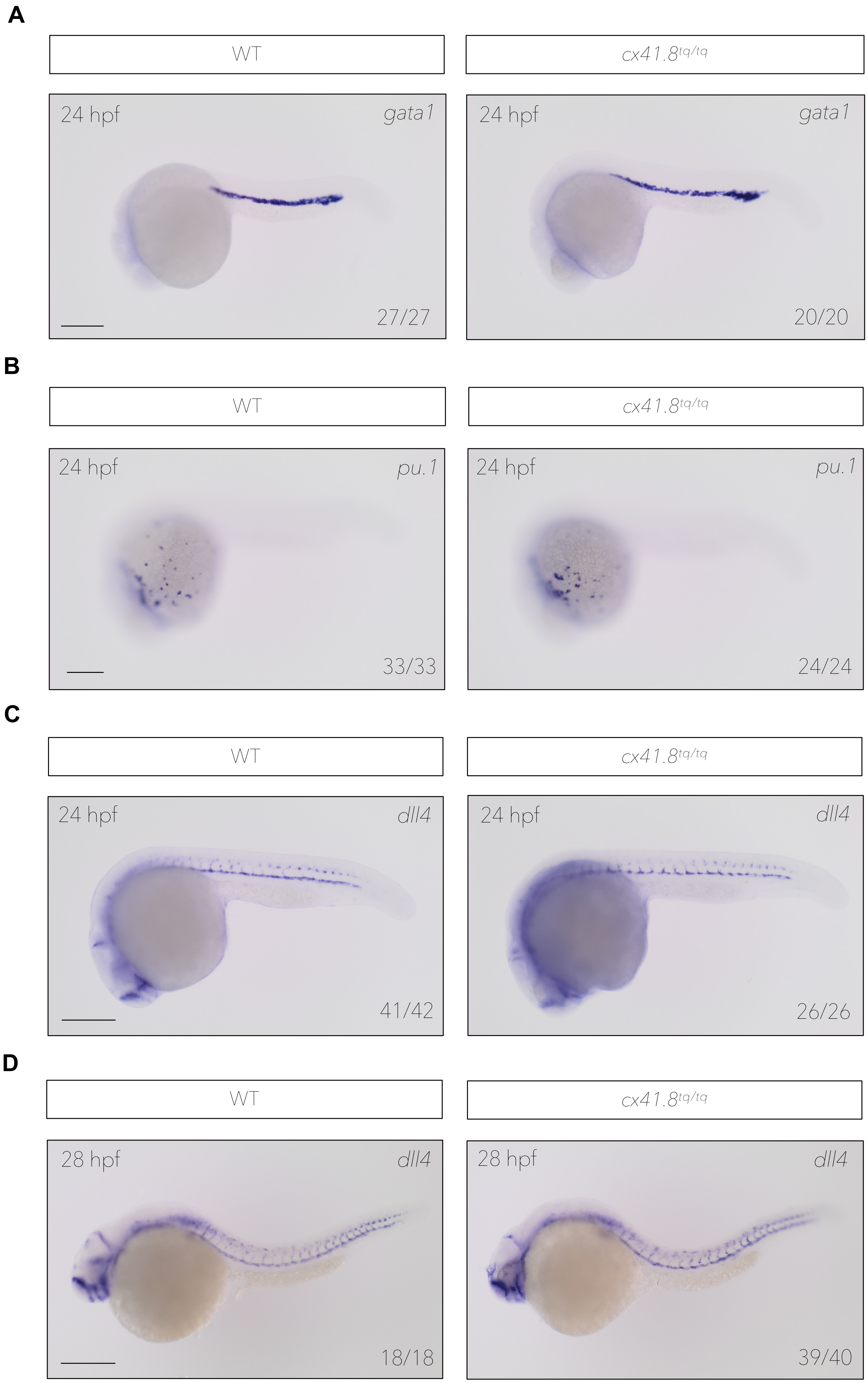

Biology Open • Supplementary information

Fig. S1. *cx41.8$^{tq/tq}$* mutant embryos do not have altered primitive haematopoiesis or vascular development

A. *in situ* hybridisation against *gata1* (primitive erythrocytes) in *cx41.8$^{tq/tq}$* mutants and controls at 24 hpf. **B.** *in situ* hybridisation against *pu.1* (primitive macrophages) in *cx41.8$^{tq/tq}$* mutants and controls at 24 hpf. **C.** *in situ* hybridisation against *dll4* (arterial endothelium) in *cx41.8$^{tq/tq}$* mutants and controls at 24 hpf. **D.** *in situ* hybridisation against *dll4* in *cx41.8$^{tq/tq}$* mutants and controls at 28 hpf. Numbers indicate the ratio of embryos with the respective phenotype. Scale bars: 200 µm (**A**, **B**, **C** and **D**).

Biology Open • Supplementary information

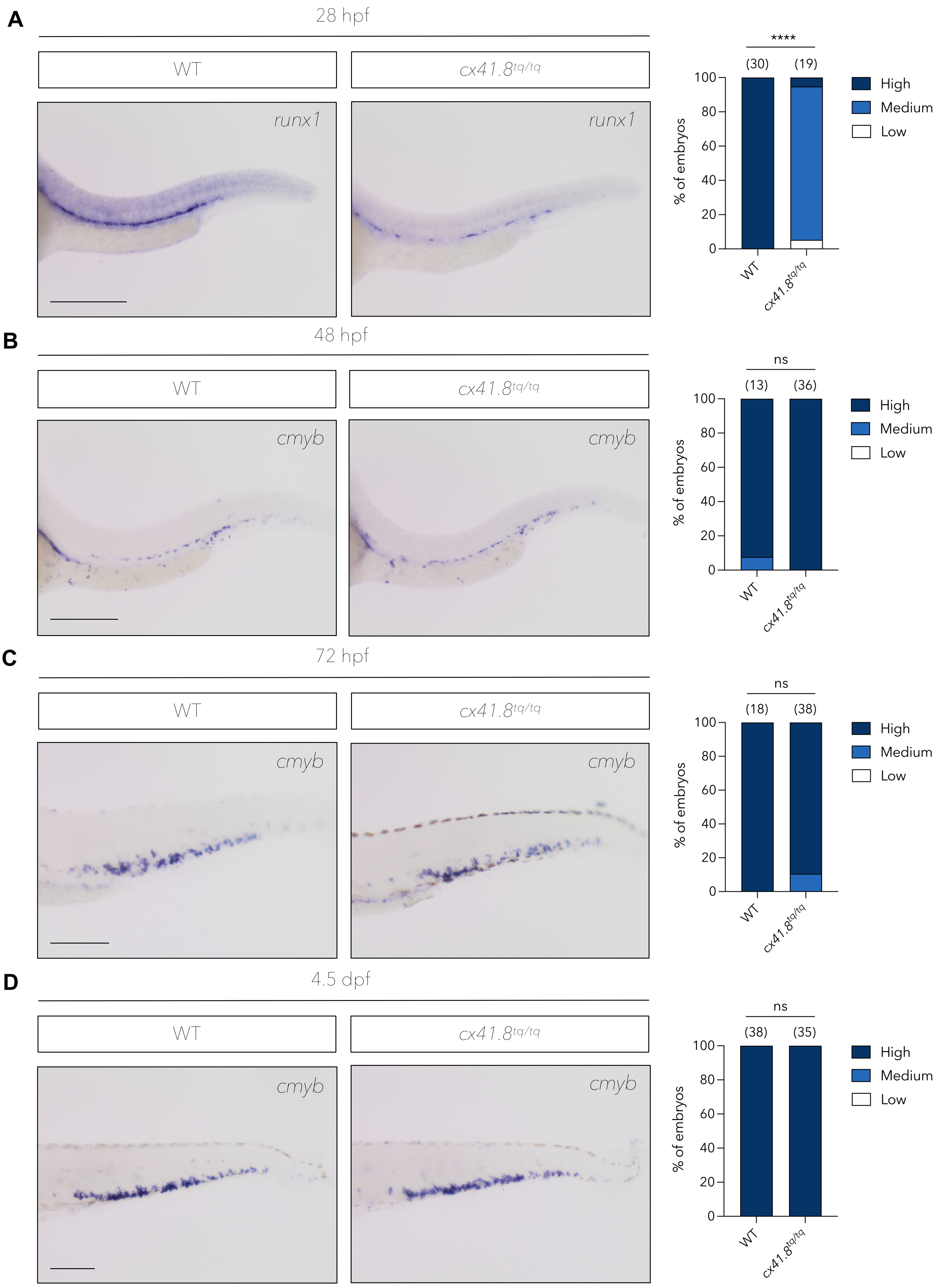

Biology Open • Supplementary information

Fig. S2. ***cx41.8**$^{tq/tq}$* **mutant embryos display a delay in HSPC specification**

A. *runx1 in situ* hybridisation and quantification in *cx41.8*$^{tq/tq}$ mutants and controls at 28 hpf. **B.** *cmyb in situ* hybridisation and quantification in *cx41.8*$^{tq/tq}$ mutants and controls at 48 hpf. **C.** *cmyb in situ* hybridisation and quantification in *cx41.8*$^{tq/tq}$ mutants and controls at 72 hpf. **D.** *cmyb in situ* hybridisation and quantification in *cx41.8*$^{tq/tq}$ mutants and controls at 4.5 dpf. Statistical significance was calculated using either a Chi-squared test (**A**) or Fisher's test (**B**-**D**). *p < 0.05, **p < 0.01, ***p < 0.001, ****p < 0.0001. Scale bars: 200 µm (**A**, **B**, **C** and **D**).

Biology Open • Supplementary information

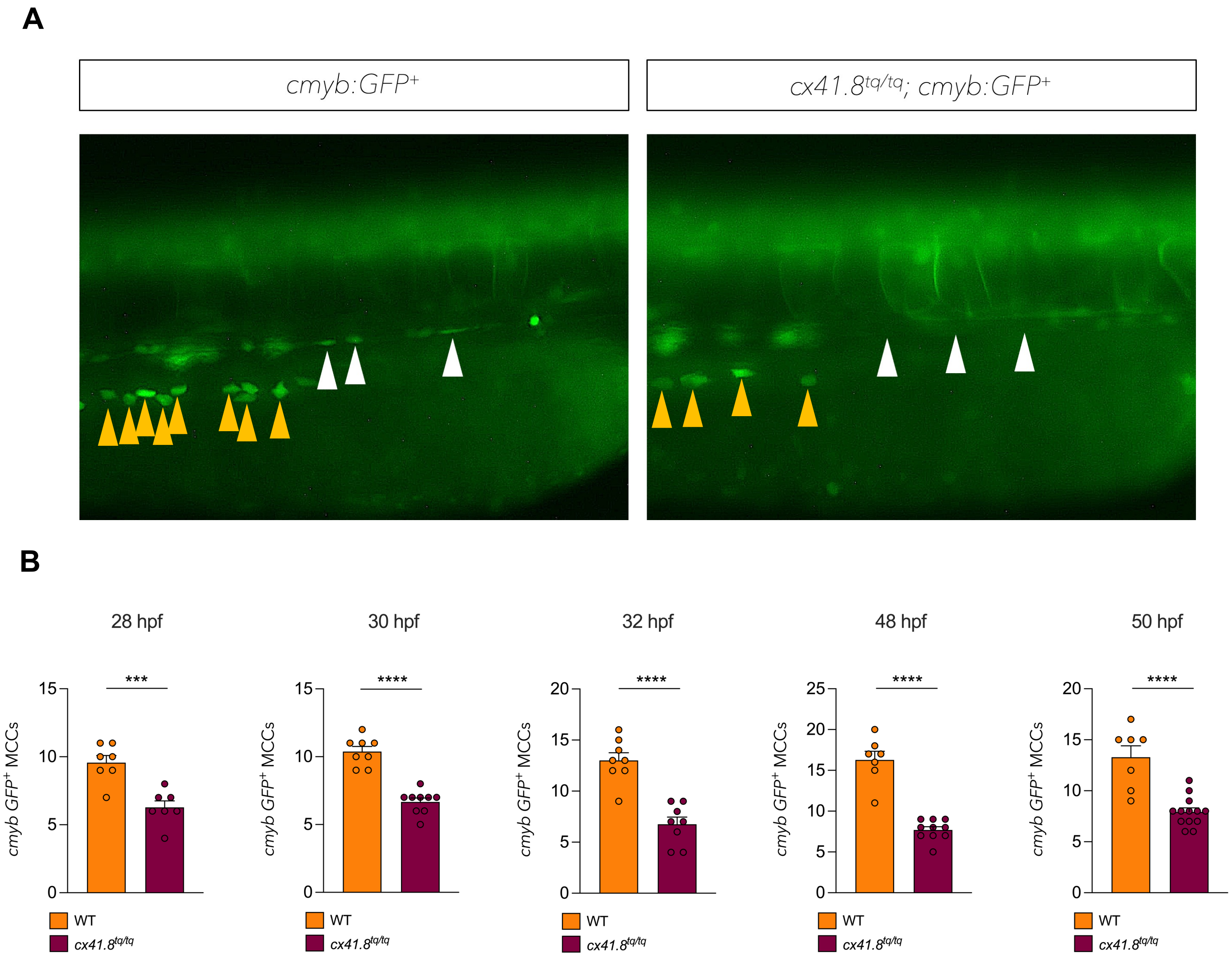

Fig. S3. ***cmyb GFP⁺*** **multiciliated cells are reduced in number along the yolk tube extension in** ***cx41.8$^{tq/tq}$*** **mutant embryos**

A. Representative image of *cmyb GFP⁺* HSPCs along the floor of the dorsal aorta (white arrowheads) and multiciliated cells along the yolk tube extension (yellow arrowheads). **B.** A reduction in *cmyb GFP⁺* multiciliated cells is present along the yolk tube extension at 28 hpf, 30 hpf, 32 hpf, 48 hpf and 50 hpf in *cx41.8$^{tq/tq}$* mutant embryos in comparison with controls. Statistical significance in **B** was calculated using an unpaired t-test.

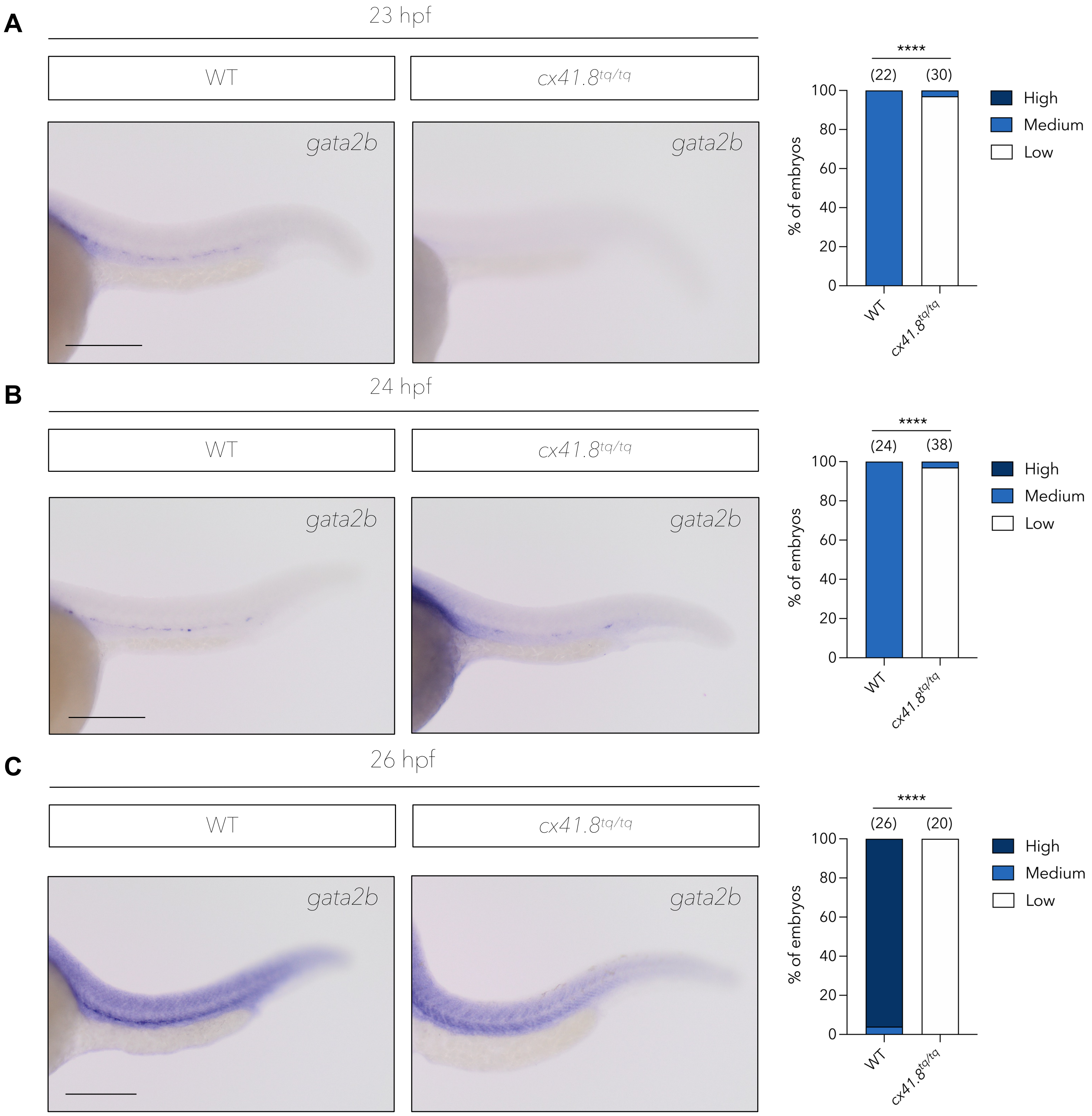

Fig. S4. *cx41.8$^{tq/tq}$* mutant embryos display a delay in *gata2b* expression

A. *gata2b in situ* hybridisation and quantification in *cx41.8$^{tq/tq}$* mutants and controls at 23 hpf. **B.** *gata2b in situ* hybridisation and quantification in *cx41.8$^{tq/tq}$* mutants and controls at 24 hpf. **C.** *gata2b in situ* hybridisation and quantification in *cx41.8$^{tq/tq}$* mutants and controls at 26 hpf. Statistical significance was calculated using either a Fisher's test (**A** and **B**) or Chi-squared test (**C**). *p < 0.05, **p < 0.01, ***p < 0.001, ****p < 0.0001. Scale bars: 200 µm (**A**, **B** and **C**).

Biology Open • Supplementary information

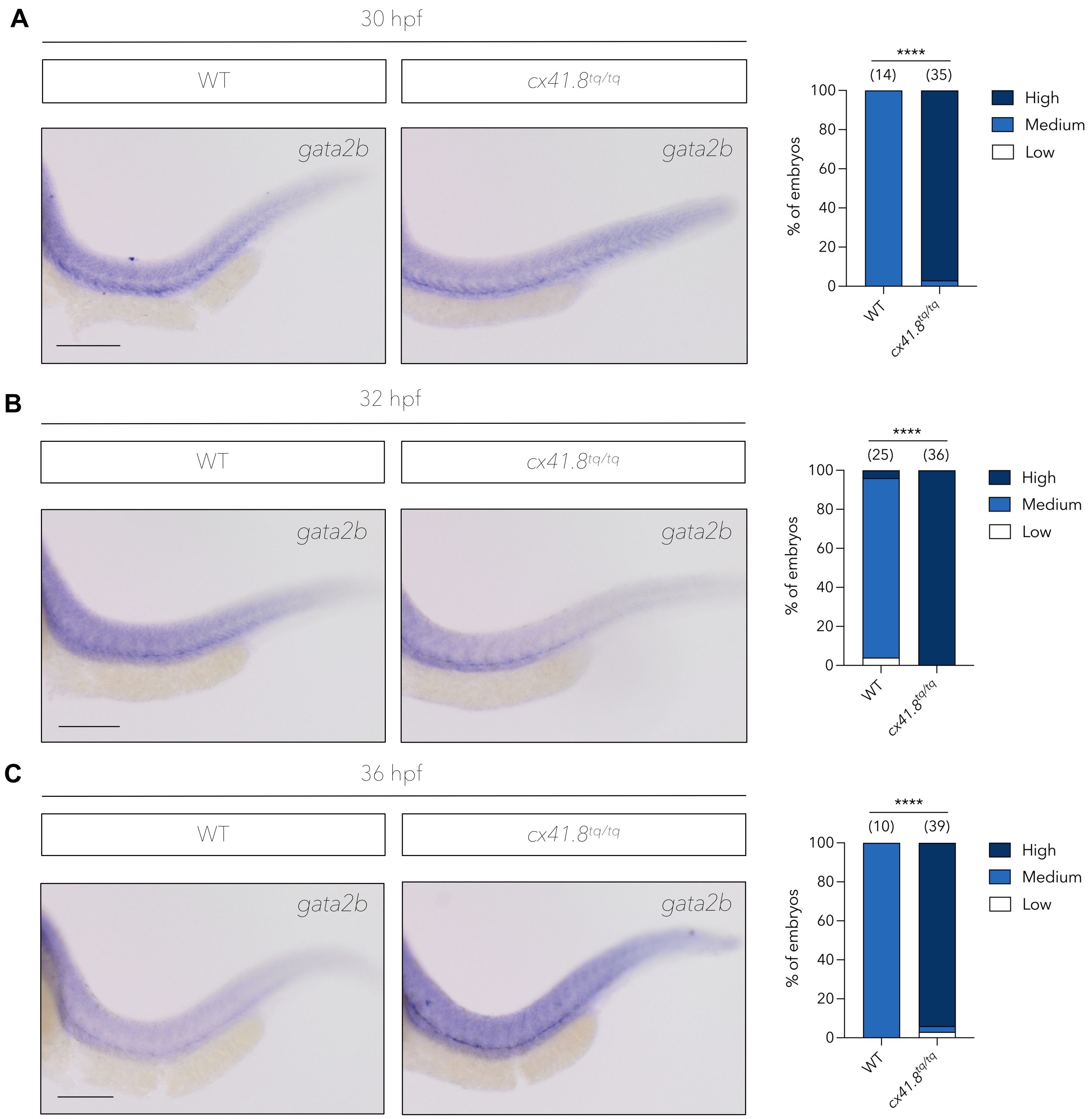

Fig. S5. ***cx41.8$^{tq/tq}$*** **mutant embryos display a delay in *gata2b* expression** **A.** *gata2b in situ* hybridisation and quantification in *cx41.8$^{tq/tq}$* mutants and controls at 30 hpf. **B.** *gata2b in situ* hybridisation and quantification in *cx41.8$^{tq/tq}$* mutants and controls at 32 hpf. **C.** *gata2b in situ* hybridisation and quantification in *cx41.8$^{tq/tq}$* mutants and controls at 36 hpf. Statistical significance was calculated using a Chi-squared test (**A**, **B** and **C**). *p < 0.05, **p < 0.01, ***p < 0.001, ****p < 0.0001. Scale bars: 200 µm (**A**, **B** and **C**).

Biology Open • Supplementary information

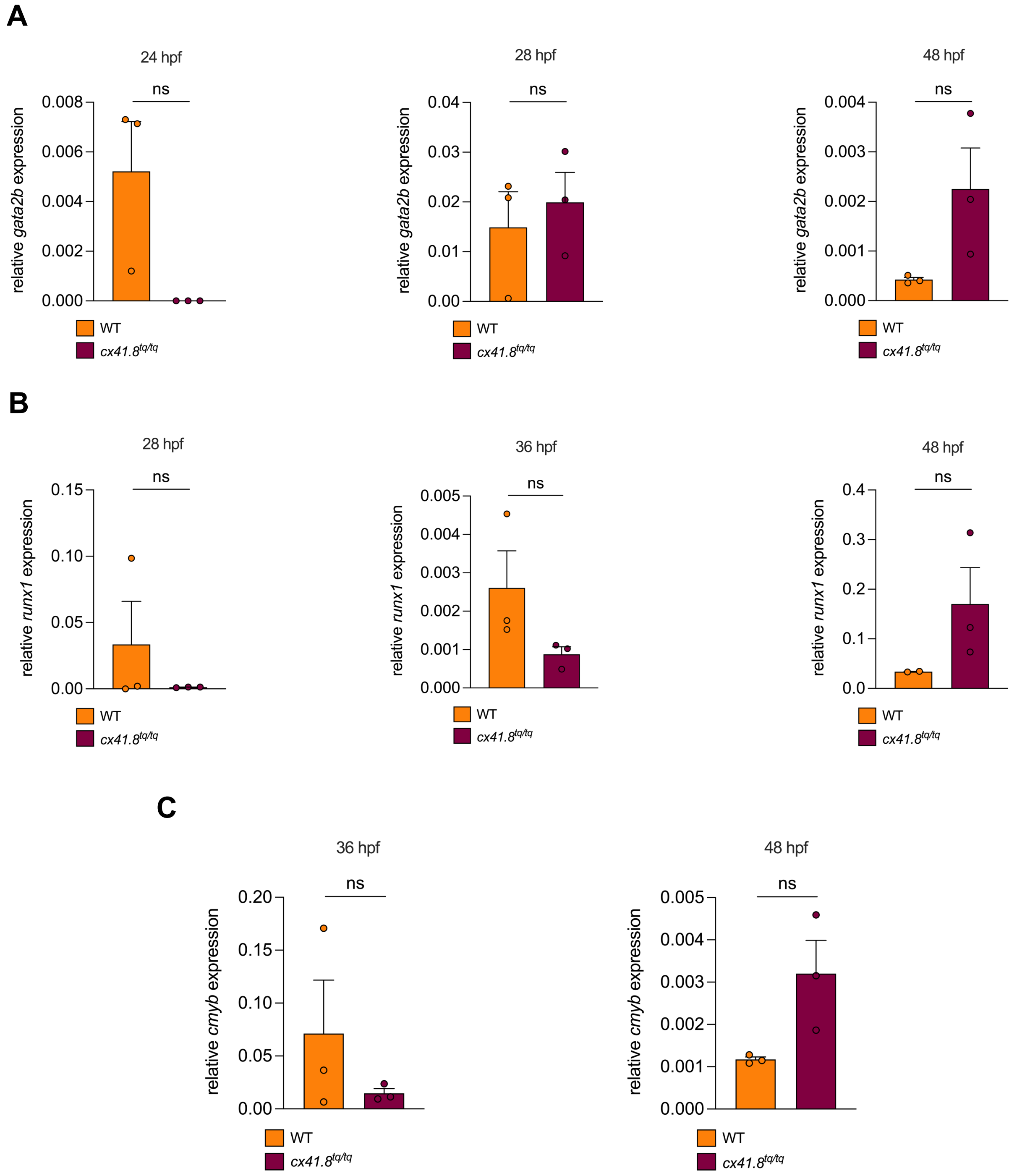

Fig. S6. *cx41.8$^{tq/tq}$* mutant embryos display a delay in *gata2b, runx1* and *cmyb* expression relative to controls

A. *gata2b* expression in trunks and tails of *cx41.8$^{tq/tq}$* mutants and wild-type controls at 24, 28 and 48 hpf, as determined by qPCR. **B.** *runx1* expression in trunks and tails of *cx41.8$^{tq/tq}$* mutants and wild-type controls at 28, 36 and 48 hpf, as determined by qPCR. **C.** *cmyb* expression in trunks and tails of *cx41.8$^{tq/tq}$* mutants and wild-type controls at 36 and 48 hpf, as determined by qPCR.

Biology Open • Supplementary information

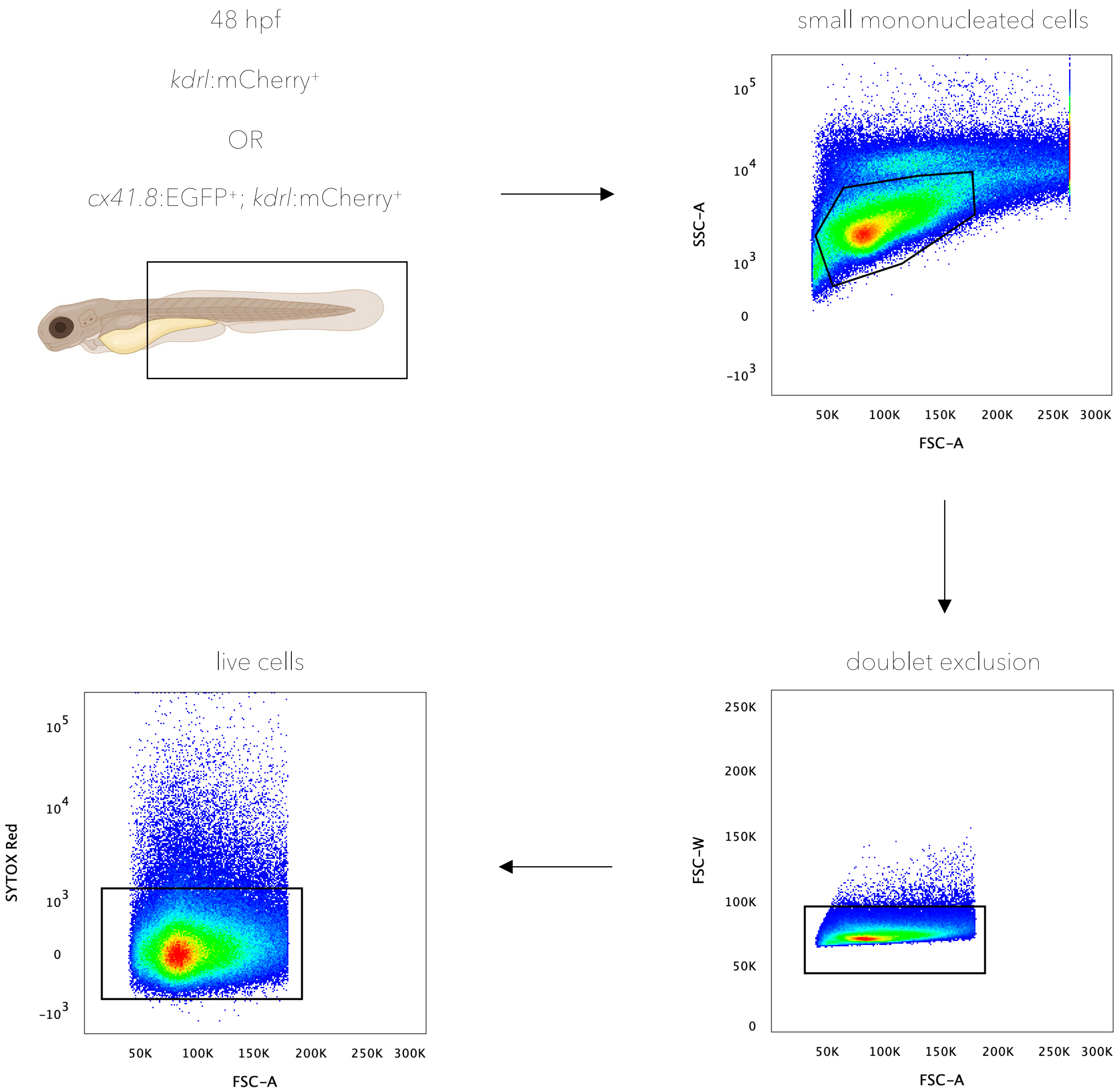

Fig. S7. Gating strategy for flow cytometry analyses

Trunk and tail dissection and flow cytometry gating strategy for 48 hpf *kdrl*:mCherry+ or *cx41.8*:EGFP+; *kdrl*:mCherry+ embryos. Created in BioRender by Petzold, T., 20205. https://BioRender.com/i9wqnnx. This figure was sublicensed under CC-BY 4.0 terms.

Biology Open • Supplementary information

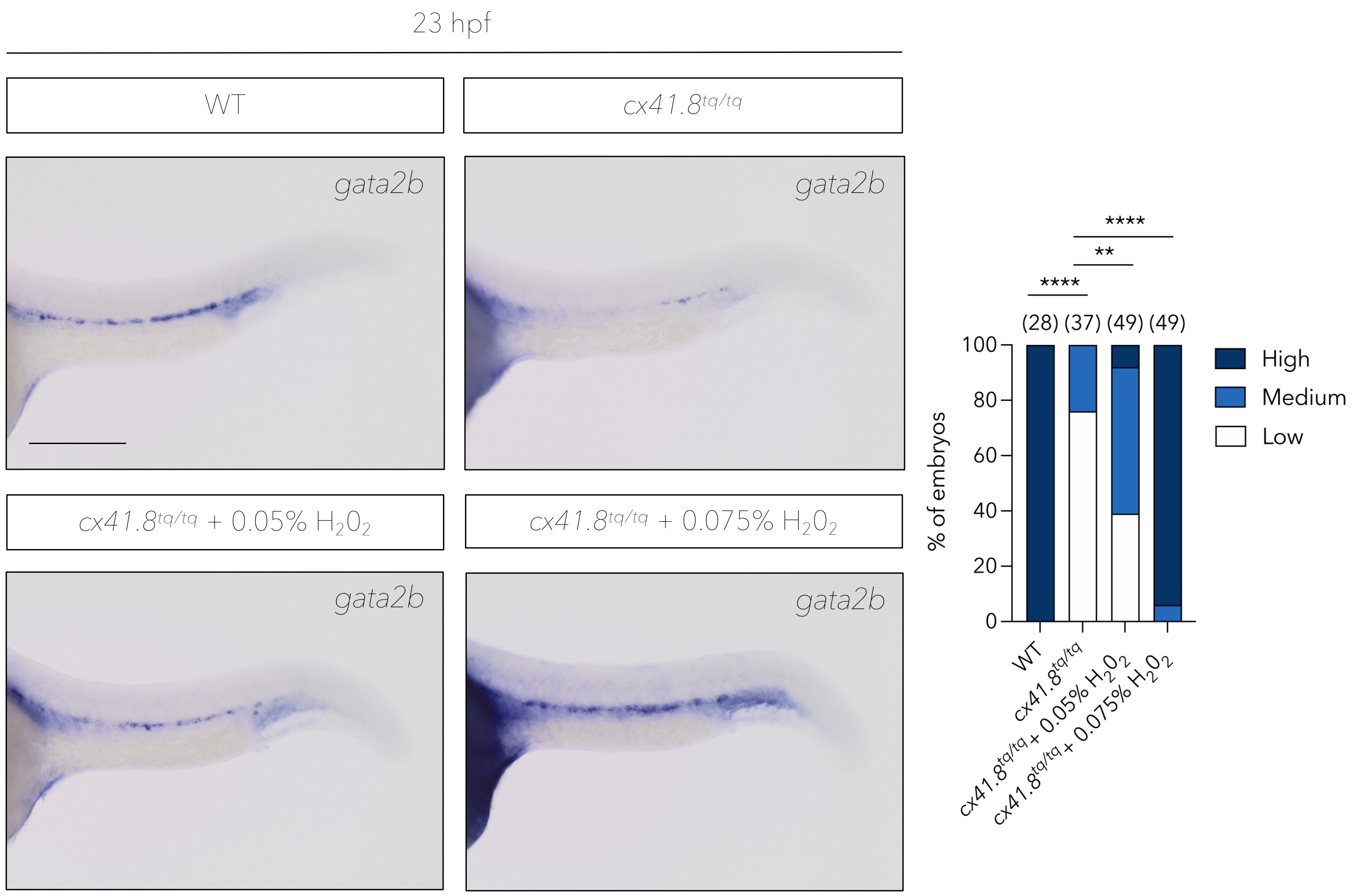

Fig. S8. ROS induction in *cx41.8$^{tq/tq}$* mutant embryos rescues *gata2b* loss in a dose dependent manner at 23 hpf

gata2b in situ hybridisation and quantification in controls and *cx41.8$^{tq/tq}$* mutants supplemented with either 0.05% or 0.075% H_2O_2. Statistical significance was calculated using a Chi-squared test. *p < 0.05, **p < 0.01, ***p < 0.001, ****p < 0.0001. Scale bar: 200 µm.

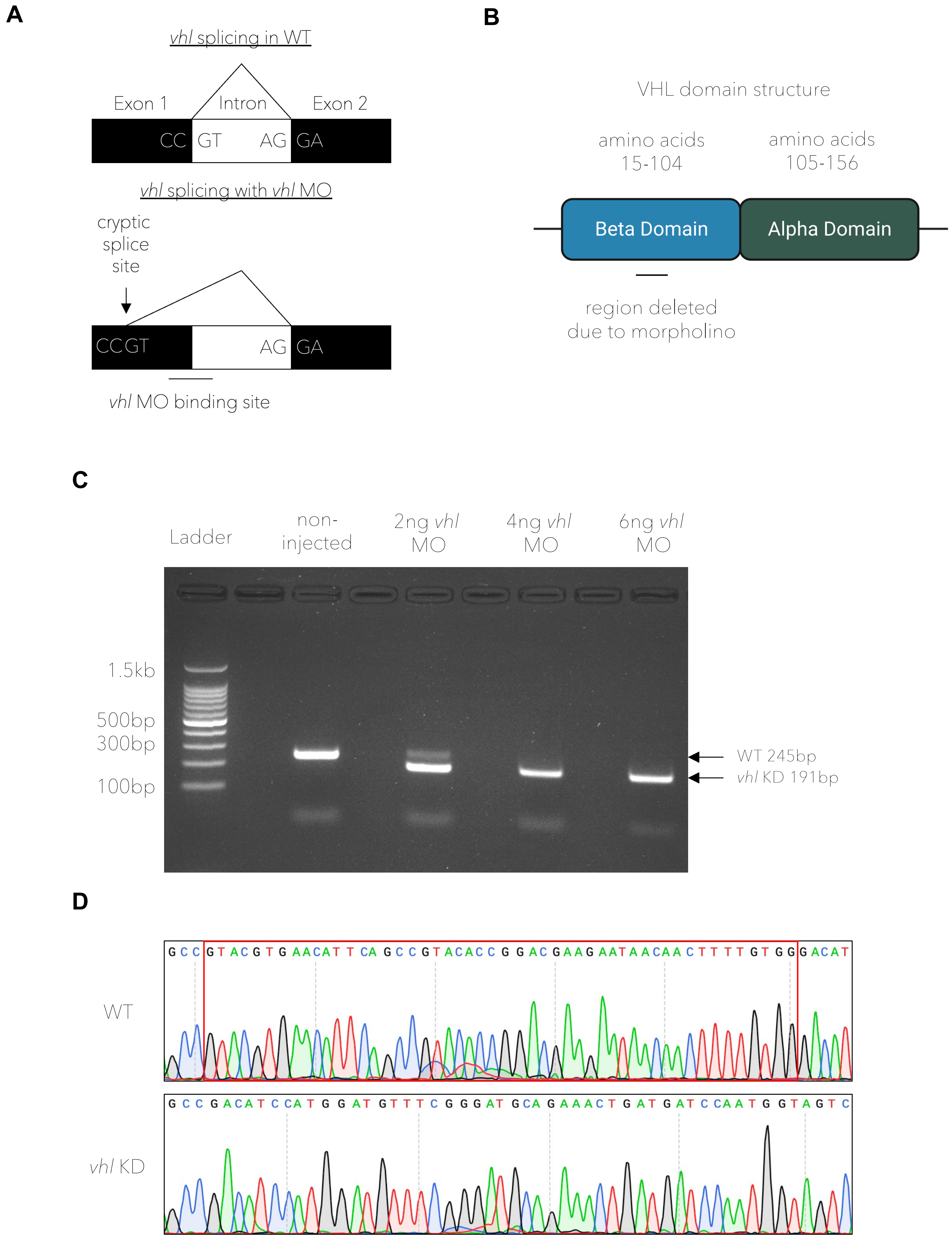

Biology Open • Supplementary information

Fig. S9. The *vhl*-MO results in the loss of 18 amino acids from the VHL beta domain

A. Schematic to show the location of the *vhl*-MO induced cryptic splice site in exon 1 of the *vhl* transcript. **B.** Schematic to show the location of the *vhl*-MO induced loss of 18 amino acids from the VHL beta domain. **C.** Gel electrophoresis image showing the *vhl*-MO induced 54bp loss in the *vhl* transcript with 6ng of *vhl*-MO. **D.** Sanger sequencing confirming the 54bp loss in the *vhl* transcript with 6ng of *vhl*-MO. The red box in the WT sequencing track indicates the 54bp lost in the *vhl* transcript upon *vhl*-MO injection.

Biology Open (2025): doi:10.1242/bio.062118: Supplementary information

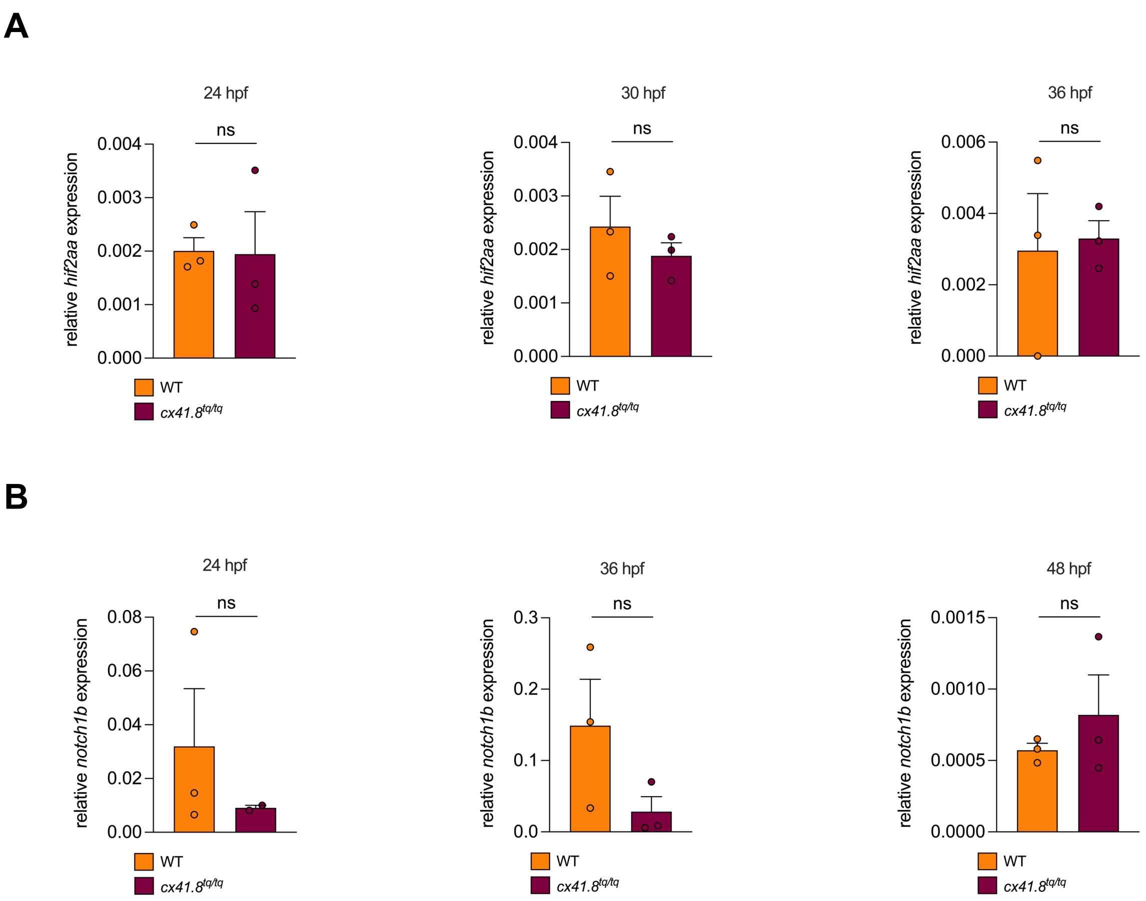

Fig. S10. *hif2aa* and *notch1b* expression in trunks and tails of *cx41.8*$^{tq/tq}$ mutant embryos relative to controls

A. *hif2aa* expression in trunks and tails of *cx41.8*$^{tq/tq}$ mutants and wild-type controls at 24, 30 and 36 hpf, as determined by qPCR. **B.** *notch1b* expression in trunks and tails of *cx41.8*$^{tq/tq}$ mutants and wild-type controls at 24, 36 and 48 hpf, as determined by qPCR.

Biology Open • Supplementary information

Table S1. Primers used for genotyping *cx41.8*$^{tq/tq}$ mutant embryos

Mutant	Forward	Reverse
cx41.8$^{tq/tq}$	TGCTGCAAACATACGTCCTC	TTTGCAGAGTTCTGCTGGTG

Table S2. Primers used for qPCR

Gene	Forward	Reverse
gata2b	ACCACCACACTCTGGAGAC	CTGTTGCGTGTCTGAATACC
runx1	CGGTGAACGGTTAATATGAC	CTTTTCATCACGGTTTATGC
cmyb	TGATGCTTCCCAACACAGAG	TTCAGAGGGAATCGTCTGCT
notch1b	TATGTTAAGCCTGCGTGGAC	AACATTCCAGGCCTTGTGTC
hif2aa	AGCTGGAAGGTTCTACACTG	TGGATGGACAATAGGTTCGC
ef1-alpha	GAGAAGTTCGAGAAGGAAGC	CGTAGTATTTGCTGGTCTCG

Table S3. Primers used to analyse the *vhl* knockdown efficiency of the *vhl*-MO

Gene	Forward	Reverse
vhl	CAGGTCAACGTTCTGTTCTG	GTGATCTTGGCATTTGCGAC

Biology Open • Supplementary information
